# Dynamic Loading of Lattice Structure Made by Selective Laser Melting-Numerical Model with Substitution of Geometrical Imperfections

**DOI:** 10.3390/ma11112129

**Published:** 2018-10-29

**Authors:** Radek Vrána, Ondřej Červinek, Pavel Maňas, Daniel Koutný, David Paloušek

**Affiliations:** 1 Institute of Machine and Industrial Design, Faculty of Mechanical Engineering, Brno University of Technology, Technická 2896/2, 616 69 Brno, Czech Republic; Radek.Vrana@vut.cz (R.V.); Daniel.Koutny@vut.cz (D.K.); David.Palousek@vut.cz (D.P.); 2 Department of Engineer Technology, Faculty of Military Technology, University of Defence, Kounicova 65, 662 10 Brno, Czech Republic; Pavel.Manas@unob.cz

**Keywords:** finite element analysis (FEA), low-velocity impact, numerical model, lattice structure, material model, ANSYS Workbench, aluminum alloy AlSi10Mg, energy absorption

## Abstract

Selective laser melting (SLM) is an additive technology that allows for the production of precisely designed complex structures for energy absorbing applications from a wide range of metallic materials. Geometrical imperfections of the SLM fabricated lattice structures, which form one of the many thin struts, can lead to a great difference in prediction of their behavior. This article deals with the prediction of lattice structure mechanical properties under dynamic loading using finite element method (FEA) with inclusion of geometrical imperfections of the SLM process. Such properties are necessary to know especially for the application of SLM fabricated lattice structures in automotive or aerospace industries. Four types of specimens from AlSi10Mg alloy powder material were manufactured using SLM for quasi-static mechanical testing and determination of lattice structure mechanical properties for the FEA material model, for optical measurement of geometrical accuracy, and for low-velocity impact testing using the impact tester with a flat indenter. Geometries of struts with elliptical and circular cross-sections were identified and tested using FEA. The results showed that, in the case of elliptical cross-section, a significantly better match was found (2% error in the *F_max_*) with the low-velocity impact experiments during the whole deformation process compared to the circular cross-section. The FEA numerical model will be used for future testing of geometry changes and its effect on mechanical properties.

## 1. Introduction

Energy absorbers made of porous materials are currently used to absorb mechanical energy caused by impact or high velocity deformation due to their high efficiency of energy absorption and low weight [1,2,3]. There are several types of commercially produced porous materials, e.g., hexagonal honeycomb structures [4], metal foams [5,6,7], or laminated composite fiber blocks [8]. Mostly, the aluminum foams are used. They usually have porosity about 75–95% with a large amount of closed gas pockets and irregular porous structure. This material is usually used in the form of sandwich panels to achieve a higher absorption effect through uniform distribution of stress during loading.

An alternative way to produce porous materials with precisely controlled shape of porous geometry is the SLM technology [9]. SLM uses a layer-based production which allows for the manufacturing of the porous material with a complex shape that can be designed directly for the expected amount of impact energy. Using SLM, it is also possible to integrate screw holes or other fixation elements to the porous material. Unlike conventionally produced materials, SLM allows the production of the porous material from various alloys such as titanium or tool steels alloys [10]. The most commonly used shape of lattice structure produced by SLM is BCC (Body Centered Cubic) [9,11]. BCC geometry corresponds to body diagonals of the cube. It consists of eight struts intersecting in its center. Orientation of the struts in BCC structure is 35.26° compared to *xy* plane.

During SLM production of the lattice structure, geometrical imperfections occur. They are caused by struts orientation and heat transfer to the surrounding metal powder. Consequently, the laser process parameter needs to be optimized for SLM production of lattice structure [12,13,14,15,16,17,18,19]. Vrana et al. [19] deal with the SLM processing strategy for strut-lattice structure production, which uses only contour lines and various combinations of main process parameters. The authors focused on the evaluation of the influence of a laser scanning strategy on material properties and surface roughness. The best results were achieved with 25% track overlapping, input energy *E_inp_* in the range from 9 J to 10.5 J and linear energy *E_lin_* from 0.25 to 0.4 J/mm; in particular, the relative density of 99.83% and the surface roughness on the side of the strut of Ra 14.6 µm in an as-built state was achieved. Geometrical imperfections are mainly shape deviations created by sticking of the partly melted powder particles onto the down skin side of struts [19,20,21], high surface roughness, and internal porosity. Sticking of powder was also dealt with by Koutny et al. [20]. These authors studied the influence of SLM production orientation on the real diameter of struts. The results show a dependence between the struts diameter and production orientation. In the case AlSi10Mg, the diameter of the struts was always larger, and their true diameter changed with orientation of the strut (compared to the platform). Qui et al. [14] also examined the influence of laser process parameters onto the strut diameter. The results show that single struts manufactured by SLM had a larger diameter than nominal. The diameter increased monotonically with higher laser power and it significantly improved compression mechanical properties of the lattice structure compared to the assumption. Similar results were achieved by Vrana et al. [22] in the case of lattice structure under low-velocity impact loading. The results from mechanical testing show a significant improvement of the impact resistance due to the strut diameter increase.

For efficient design of energy absorber, it is necessary to use FEA to predict mechanical properties of the part during impact load. There are two main approaches to the numerical models of porous materials. The former uses a homogenized model of geometry and the latter uses a simplified model of real geometry [2,4,23,24,25,26,27]. The method of how to simplify the real shape of the lattice struts for FEA was described by Suard et al. [21]. They studied the shape of the lattice structure struts produced by EBM technology. A Computed tomography (CT) analysis was used for a detailed 3D scan of the strut surface. For geometry simplification in FEA, the effective volume corresponding with the maximum cylinder inscribed in the strut was defined. Koutny et al. [20] measured the shape of struts specimens using optical measurement. Similar to the previous author, the maximum inscribed diameter was used for the evaluation of mechanical properties.

Porous materials have a specific impact loading behavior due to the topology of core geometry. Therefore, in the case of homogenized geometry, it is necessary to use a suitable material model that considers its deformation behavior. Material models of porous structures, such as honeycomb or metal foam, are usually included in the material library of the FEA software, and it is possible to also use them for lattice structure [10,26,27,28]. According to Mohmmed et al. [26], a crushable foam material model is suitable for simulation of penetration of porous foam blocks with a damage criterion describing the occurrence of breakdowns between the core and plates. Input material constants can be obtained from uniaxial compression tests according to ASTM D5308. Labeas et al. [27] used both ways; the material model Mat-26 Honeycomb (LS-Dyna) to create a dynamic FEM simulation with a homogenized micro-lattice core and the bilinear (multilinear) material model with micro-lattice BCC structure geometry. The results showed that the simplified core is only suitable for prediction of the first progressive collapse of the lattice structure, while the beam geometry allows for the prediction of the whole deformation process due to the preserving topology of the core. Based on previous studies [10,26,27], it is possible to determine boundary conditions, type and density of polygonal mesh, type of contact between bodies. It is necessary to consider the difference between the core and plate material model and the damage criterion [19,24,29,30] that needs to be added.

The authors [31,32,33] examined mechanical properties of AlSi10Mg alloy produced by SLM technology. As tensile specimens, the standard or flat specimens in the as-build or machined condition were usually used. Kempen et al. [33] showed various mechanical properties depending on the SLM production orientation. Specimens with *xy* orientation achieved a higher elongation compared to the z direction. The influence of the strut shape and SLM process parameters was dealt with by Tsopanos et al. [34]. In their study, the single struts from 316L alloy were tested. The results showed significant differences between the mechanical properties of struts with internal porosity or non-melted particles compared to the well melted struts. It is caused by small dimensions of struts compared to the standard tensile specimens. Therefore, special multi-strut tensile specimens were designed in this study.

Porous materials as honeycomb or metal foams are already used as a highly effective absorber in industry. Currently, metal additive technologies such as SLM can be used as one of the ways for production of energy absorbers. Thanks to the additive production, it is possible to customize the absorbers for specific impact loading by the structure shape design (various areas with a different type of structure, gradient structure [35], etc.) or by the used material. SLM technology also has a few technological limitations that should be considered in FEA. In the case of thin struts production, small shape deviations can occur. Due to the high number of the struts inside the lattice structure, these imperfections can influence mechanical properties of whole structure. Therefore, this study deals with the influence of SLM technology imperfections during struts production and their mechanical response in FEA.

## 2. Materials and Methods

### 2.1. Speciments Fabrication Using SLM

**Figure 1:**
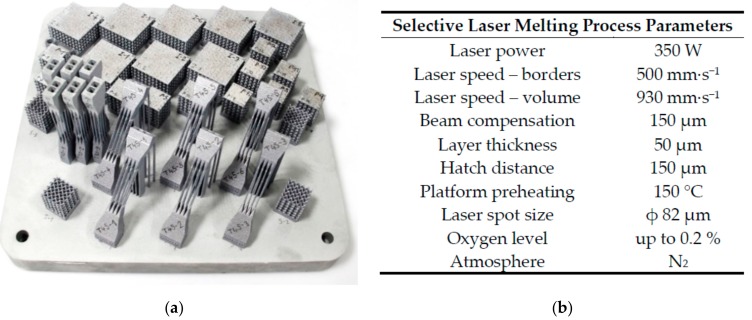
(**a**) Single series of mechanical specimens after SLM manufacturing; (**b**) SLM laser process parameters used for specimen fabrication.

All sets of specimens were manufactured using SLM 280HL machine (SLM Solutions GmbH, Lübeck, Germany) which is equipped with a 400 W Ytterbium fiber laser (YLR-laser) with Gaussian shape of energy distribution and spot diameter 82 µm. Laser scanning speed may reach up to 10.000 mm·s^−1^. During SLM process, the N_2_ atmosphere was used in a chamber which provides 280 × 280 × 350 mm build envelope. To ensure the same conditions during the manufacturing process, each set of specimens were produced in one build job (Figure 1a). Standard process parameters (SLM Solutions) were used (Figure 1b).

### 2.2. Metal Powder Analysis

**Figure 2:**
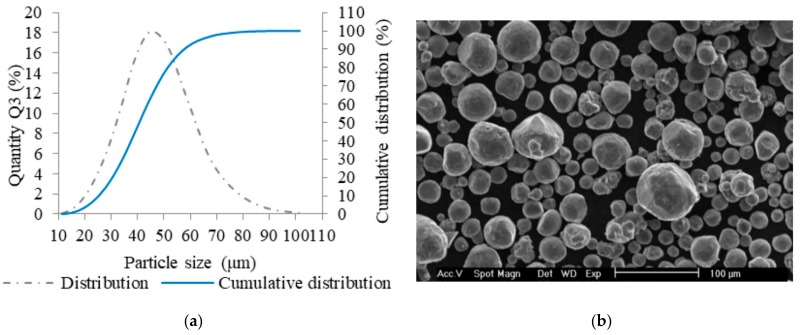
Selective laser melting (SLM) powder characteristics; (**a**) chart of particle size distribution; (**b**) shape of powder particles (scanning electron microscopy (SEM)).

AlSi10Mg aluminum alloy powder (TLS Technik GmbH, Bitterfeld, Germany) was used for manufacturing all types of specimens. The powder material with almost spherical shape of particles was produced using a gas atomization technology in nitrogen atmosphere (Figure 2b). For quality verification, the particle size distribution was analyzed (Horiba LA–960, Horiba, Kyoto, Japan). Main parameters of the particle size distribution were as follows—median size was 40.7 µm, mean size was 41.4 µm, and standard deviation was 12.9 µm. The particle size up to 25.2 µm represents 10% and the particles up to size of 58 µm represents 90% of particles (Figure 2a). Depending on the particle size distribution of the metal powder, a 50 µm layer was used for fabrication of all specimens.

### 2.3. Specimens for Mechanical Testing

#### 2.3.1. Tensile Specimens

**Figure 3:**
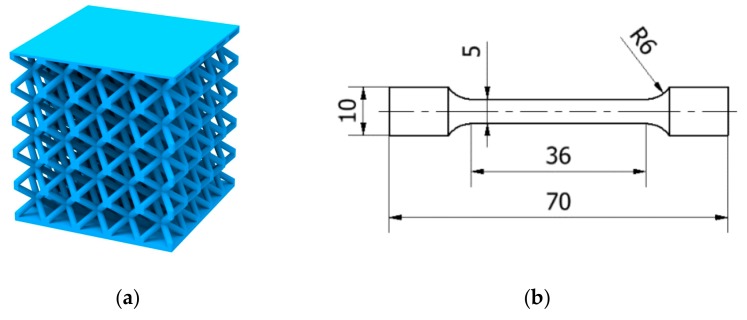

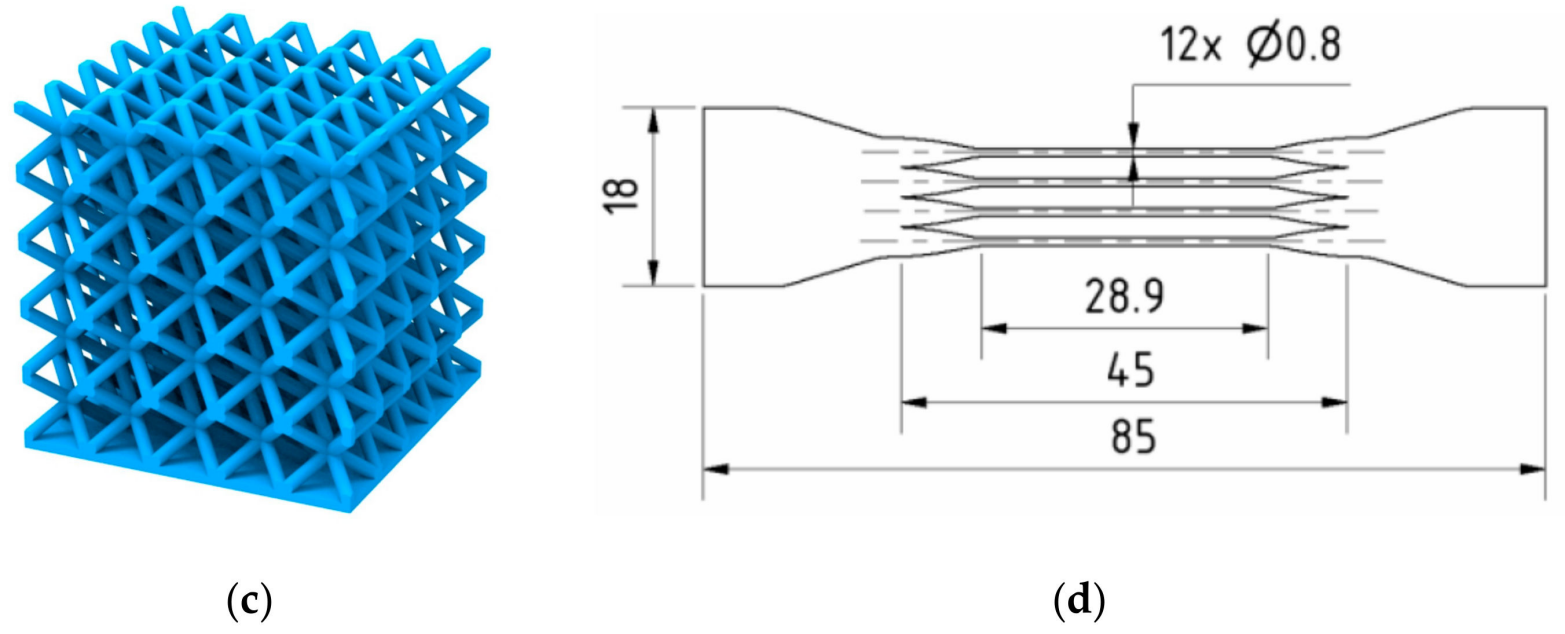
Specimens for (**a**) quasi-static compressive (C-series) and low-velocity impact testing (IT-series); (**b**) quasi-static tensile testing of bulk material (TB-series); (**c**) optical analysis (O-series); and (**d**) quasi-static tensile testing of multi-strut specimens (TS-series).

Mechanical properties of thin struts are highly affected by surface roughness and internal material porosity, which locally reduces the strut cross-section and mechanical properties [34]. Therefore, a special (multi-struts) shape of tensile specimens was designed for quasi-static mechanical testing (TS-series; Figure 3d). The multi-strut specimens were composed of 12 struts with diameters of *d* = 0.8 mm and strut lengths of *l* = 29 mm. To describe the material properties depending on specimen’s inclination during SLM layer-based fabrication, they were fabricated in orientation of 90° and 45° (relative to the platform). To compare the struts and bulk mechanical properties, standard bulk material specimens (TB-series; Figure 3b) were also fabricated in orientation of 90° and 45° (relative to the platform). All specimens were tested in the as-build condition.

#### 2.3.2. Lattice Structure Specimens

For quasi-static compression tests, BCC lattice structure core specimens with dimensions of 20 × 20 × 20.8 mm were used (C-series; Figure 3a). The BCC unit cell was composed of eight struts with diameter *d* = 0.8 mm and side length *a_BCC_* = 4 mm. On the bottom and upper side, the specimens were covered with thin plates *t* = 0.3 mm. For low-velocity impact testing, a specimen with dimensions of 20 × 20 × 16.8 mm and the same shape of the unit cell was used (IT-series; Figure 3a). To verify the material model based on parameters obtained from quasi-static testing, specimens for low-velocity impact testing with diameters of 0.6, 0.8, 1.0, and 1.2 mm were produced. Specimens for optical measurement were similar to the specimens for mechanical testing but manufactured without the upper plate for better access to the lattice structure core during the optical measurement process (O-series; Figure 3c).

### 2.4. Shape of the Struts Analysis

To determine the actual dimensions of BCC lattice structure and multi-strut tensile specimens, O-series and TS-series of the specimens were analyzed by ATOS Triple Scan (GOM GmbH, Braunschweig, Germany) optical 3D scanner (MV170 lens; calibration was carried out according to VDI/VDE 2634, Part 3). Before the scanning process, specimens were coated with a thin layer of titanium dioxide powder (approx. 3 µm) [36]. Due to the complex shape of specimens, only four-corner struts could be digitized in the required quality.

**Figure 4:**
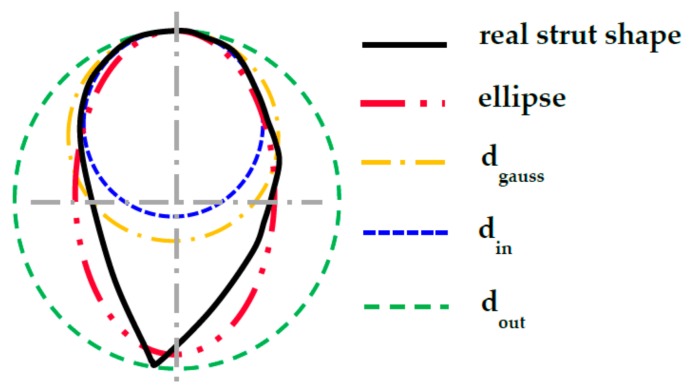
Visual 2D representation of elements used for dimensional struts analysis.

The actual dimensions were measured by fitting the ideal cylinders and ellipses into the surface geometry in GOM Inspect software (SR1, GOM GmbH, Braunschweig, Germany, Figure 4)—diameter *d_in_* (inscribed cylinder) shows the largest diameter of homogeneous strut without geometrical imperfection and surface roughness; diameter *d_out_* (circumscribed cylinder) defines the strut diameter including surface roughness and partially melted powder on the down skin strut surface; diameter *d_gauss_* shows the value with the Gaussian distribution.

To include the partially melted powder on the down skin side to the strut geometry, the ellipse geometry, which very well reflects the real shape of the strut cross-section, was used. Ellipse dimensions were measured in three points on the single corner struts, and the average value was used. Measured diameters were used for dimensional analysis of the lattice structure and for the creation of real lattice structure geometry in FEA.

### 2.5. Mechanical Testing

#### 2.5.1. Quasi-Static Mechanical Testing

Zwick Z020 device, (Zwick Z020, ZwickRoell GmbH & Co. KG, Ulm, Germany) a universal machine for mechanical testing with maximum force of 20 kN, was used for tensile (TS-series, TB-series) and compression test (C-series). Specimens were pre-loaded with 20 N and loaded with standard loading speed of 2 mm·min^−1^. During tensile testing, specimens were clamped into the jaws and loaded until all struts were broken. 

**Figure 5:**
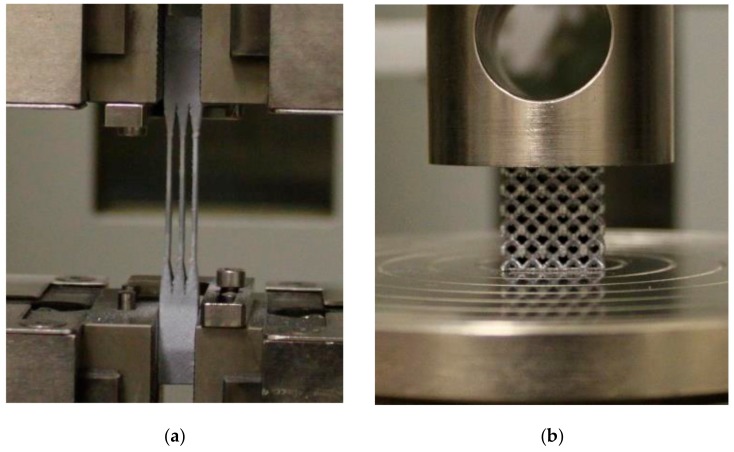
Mechanical testing using Zwick Z020 machine (**a**) tensile test; and (**b**) compression test.

During the pressure testing, the samples were placed between two plates in the testing device. The bottom plate was fixed attached to the device, thereby, movement of the sample in the vertical axis or its rotation was avoided. The upper movable plate was hinged with a rotary joint. This type of connection allowed a slight rotation of the upper (loading) plate during contacting with the sample’s surface. This eliminates the possible effect of uneven loads caused by inclined grinding of the sample surface (Figure 5).

#### 2.5.2. Low-Velocity Impact Test

**Figure 6:**
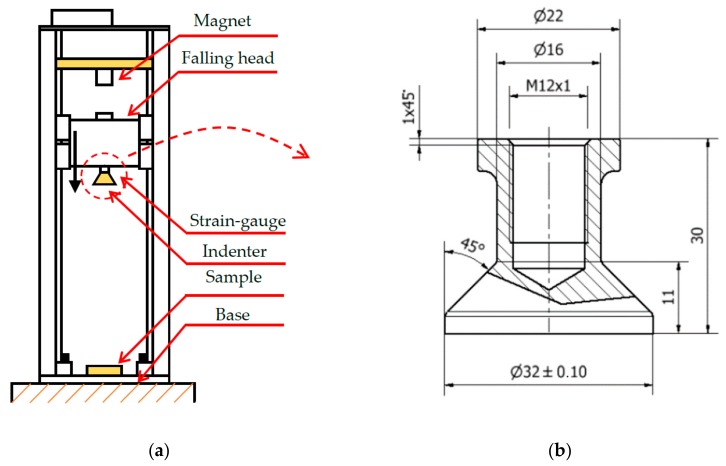
(**a**) Schema of the low-velocity impact tester; and (**b**) Geometry of the flat indenter.

Low-velocity impact testing of the IT-series was performed on the drop weight impact tester developed at Brno University of Technology (Figure 6a). The system is equipped with high-speed camera Phantom V710 and strain-gauge (XY31-3/120). The strain-gauge measures the reaction force during deformation of the lattice specimens, the high-speed camera measures the position of the marker on the falling head. Signals from the strain gauge were recorded using the data acquisition system Quantum X MX410B (HBM GmbH) with a sampling frequency of 96 kHz, data from the high-speed camera were recorded in Phantom software with a sampling frequency of 48 kHz. Both records were jointly evaluated in MATLAB software. The main output of measurements are the following dependencies: Force reaction, time (deformation), velocity of falling head, time (deformation), maximum specimen deformation, and deformation duration. The device allows to change the shape of impact body—flat indenter (surface contact; Figure 6b) and ball indenter (point contact; *d* = 16 mm). During impact testing, the weight of the falling head was *m* = 7.252 kg and the drop height was *h* = 1 m. For these parameters, the falling head achieves the maximum drop speed *v_In_* = 3.2 m·s^−1^ with maximum energy *E_In_* = 71.1 J. The testing device belongs to the group of low-velocity test devices [7,25,26].

### 2.6. FEM Numerical Model

**Figure 7:**
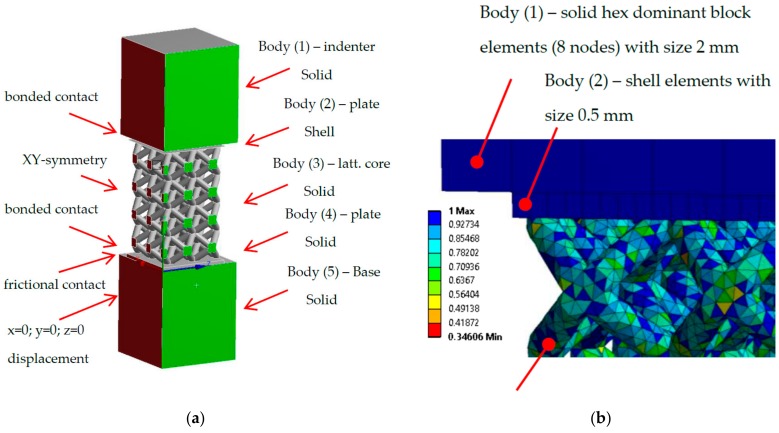
Numerical model in the Ansys software (**a**) quarter model with bodies and constrains; (**b**) finite element mesh quality.

The numerical model of the low-velocity impact test was created in ANSYS Workbench 18.2 software, module Explicit dynamic. Based on previous studies [2,4,23,24,25,26,27], the material model Bilinear isotropic hardening was selected for definition of mechanical properties of lattice core. The geometry was composed of five bodies according to Figure 7a, where the body (3) represents the lattice structured core; bodies (2) and (4) represent bottom and upper plates of the specimen; the body (1) is the indenter, and the body (5) is a solid base.

The initial drop weight impact test was performed to find out the strain rate values for various struts diameters. The obtained results were in range of 80–120 s^−1^. Based on the initial results along with the loading velocity of about of 3 m∙s^−1^, the elastic-plastic material model was selected. This model did not further consider sensitivity in the strain-rate effect.

Input parameters for definition of lattice structure core material model were determined from quasi-static tensile and compression tests of the specimen TS- and C-series, specifically from stress-strain curves, which were created based on force—displacement testing data and the geometry results from optical measurement of the specimens (see Section 3.3.1). Mechanical parameters of plates were determined from the tensile test of bulk material (TB-series). The material model was also supplemented with the criterion of damage obtained from the lattice quasi-static compression test. The used limit value corresponds with strain at the maximum stress point (*ε_σmax_*) before the progressive collapse of the lattice structure. For the indenter and the base body, the standard Structural Steel material model was used in the case of the indenter with rigid behavior.

Numerical model constrains were based on a quarter symmetry in *x* and *y* directions. From the bottom to the top in Figure 7a, between the base (5) and the bottom plate (4), the frictional contact with static frictional coefficient (0.61), and dynamic frictional coefficient of 0.47 were defined. The bottom and upper plates (4, 2) are connected with the lattice core (3) by the bonded contacts. Body self-interaction was involved. To achieve a comparable result with the experiment, only the base body (5), which represents the base plate in the testing device, was limited in *x*, *y*, *z* direction (rotation was not suppressed). To define the boundary conditions, parameters of the low-velocity impact experiment were used. The falling head (*m* = 7.25 kg) was represented by the indenter in the numerical model. As in reality, the weight of the indenter is very low compared to the falling head; therefore, the weight of the indenter was increased using a higher density value (*ρ_Ind_* = 899,306 kg·m^−3^) to match the weight of the real falling head. The impact velocity was determined using high-speed camera *v* = 3.1 m·s^−1^. For all bodies, the standard gravitational acceleration g = 9.806 m·s^−2^ was adopted.

**Figure 8:**
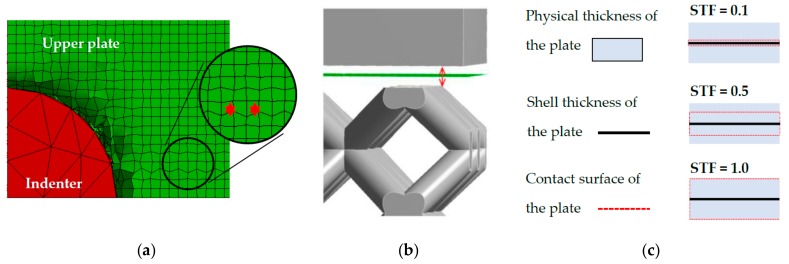
(**a**) Hour-glassing energy error; Shell thickness factor—(**b**) Shell mid-surface of the upper plate; and (**c**) Description of the contact surface.

A finite element mesh was created with several element types (Figure 7a)—the base and indenter bodies (1, 5) were formed by Hex dominant block elements (8 nodes) with size 2 mm, the bottom plate (4) with Hex Dominant block elements (8 nodes) with a size of 1 mm, the lattice core (3) with solid Tetrahedron (4 nodes) elements, which also well represents the surface roughness of the struts (Figure 7b). Their size was managed by the diameter of struts and the mesh quality parameter. In the case of circular cross-section shape with diameter *d* = 0.95 mm, tetrahedron element size was 0.4 mm. The shell elements with size of 0.5 mm were used for upper plate (2) to prevent the Hourglass effect (Figure 8a). 

In the case of a mid-surface representation, all physical and geometrical information are represented only by the surface of shell elements without thickness (Figure 8b). For the correct physical representation and constrain application between the upper plate and indenter, the shell thickness factor was considered and set to *STF* = 0.95. This parameter ensures a contact surface in real distance from the mid-surface (Figure 8c).

## 3. Results

In presented study, there are a lot of used abbreviations, therefore, the table which summarizes them was created (Table 1).

### 3.1. The Analysis of Initial Weight and Height

After SLM fabrication, the basic parameters, such as weight and height of C-series, were carried out (nominal struts diameter *d* = 0.8 mm). The results showed that the weight of the specimens was almost twice as high and the relative density $\bar{\rho }$, which was found comparing the real weight and the theoretical weight of the solid cube, was about 10% higher than that expected by CAD. Therefore, the lattice structure numerical model must have struts diameter larger than the nominal diameter *d* = 0.8 mm. The deviation was caused by SLM production of larger struts of the lattice structure, as was also described in the study in Reference [14]. Based on these results (Table 2), more detailed analyses using optical measurement were performed.

### 3.2. Optical Measurement of the Lattice Structure

**Figure 9:**
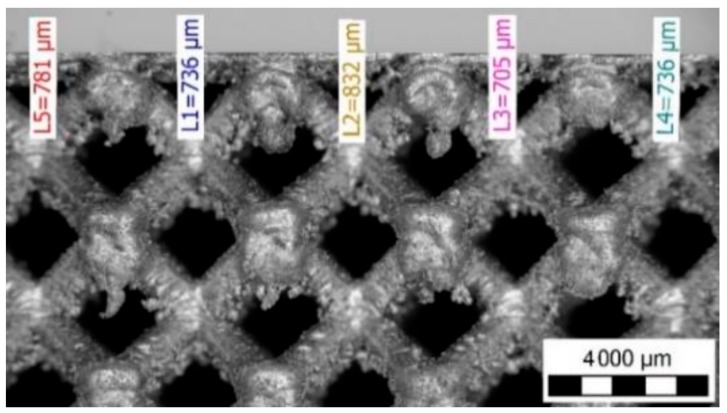
Side view on the C-series specimen using the lighting microscope.

The optical system Atos Triple Scan III (GOM GmbH, Braunschweig, Germany) and the lighting microscope Olympus SZX7 (Olympus, Tokyo, Japan) were used for more detailed measurements of the lattice structure. The result shows that there were significant differences between the inscribed and circumscribed cylinders (Table 3, Figure 4 and Figure 9).

### 3.3. Mechanical Properties

#### 3.3.1. Quasi-Static Mechanical Testing

**Figure 10:**
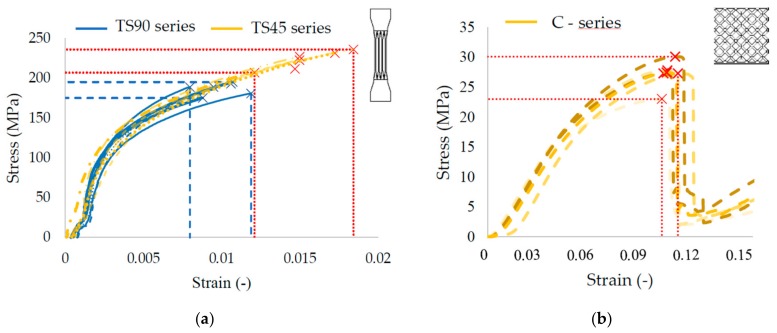
(**a**) Quasi-static stress-strain curves of the struts tensile specimens; and (**b**) Quasi-static stress-strain curves of the compression specimens.

For evaluation of mechanical properties, the average dimensions of *d_gauss_* were used (Table 4; Figure 10). From the stress-strain curves, yield strength *YTS_0.2%_*, Young’s Modulus *E*, and tangent modulus *E_T_* were evaluated. *YTS_0.2%_* was carried out as an intersection of the stress-strain curve and the parallel line to the linear part of the curve (Hook area) in the strain value *0.002*. *E_T_* tangent modulus was obtained as an interpolation of the part of the plastic area in a stress-strain curve by a line. The same evaluation process was used in the case of bulk material specimens (TB-series). The obtained average values are shown in Table 5.

#### 3.3.2. Low-Velocity Impact Test Results

**Figure 11:**
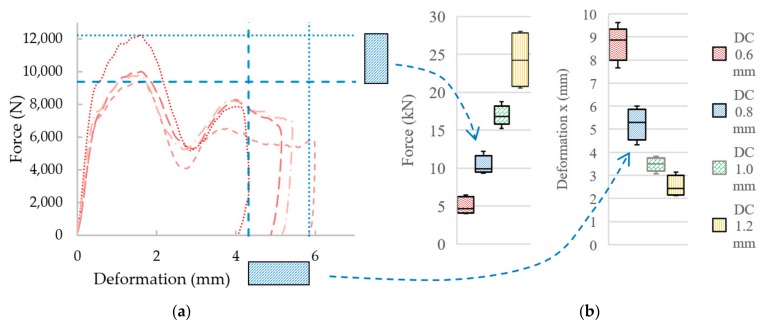

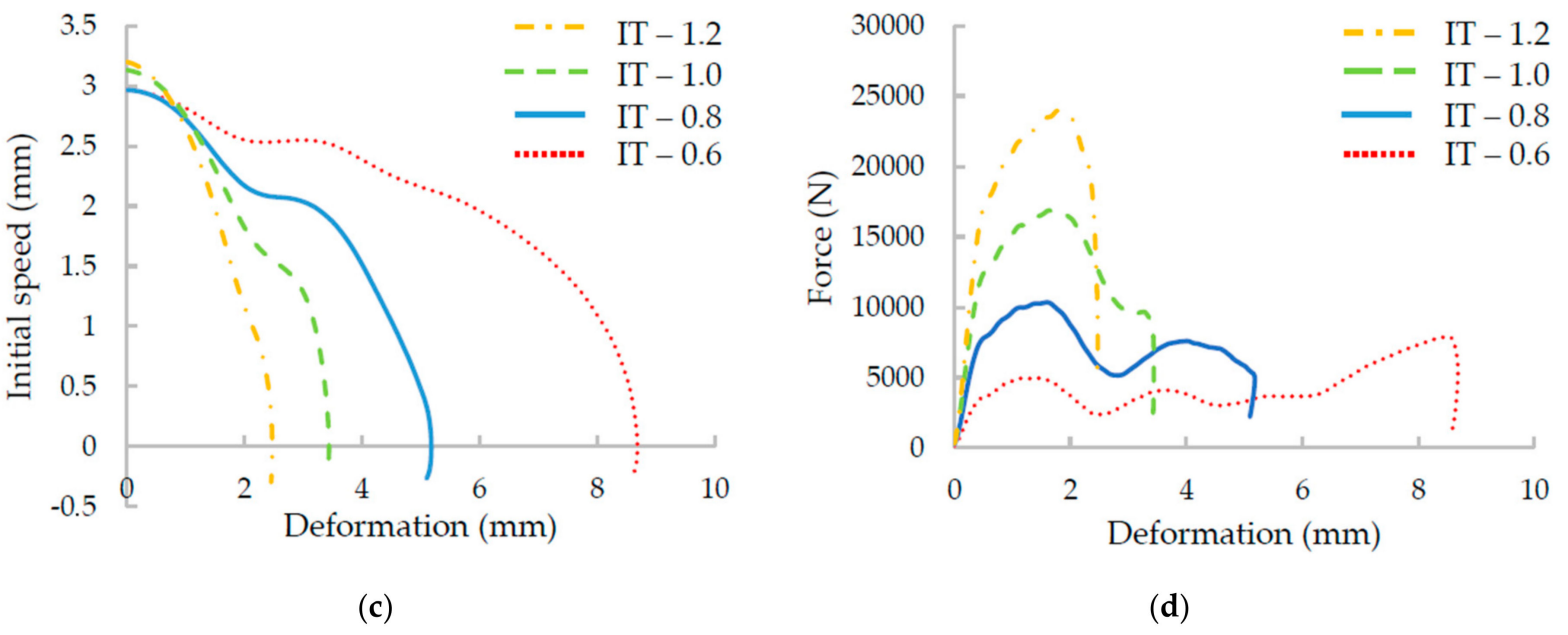
The results from low-velocity impact testing: (**a**) Single IT-series with diameter *d* = 0.8 mm; (**b**) variance of force and deformation of all IT-series; (**c**) average initial speed, deformation curves; and (**d**) average force-deformation curves.

To find out the absorption characteristics of the BCC lattice structure material and FEA for validation, the low-velocity impact test of the IT-series was carried out using the low-velocity impactor. As was described above, the specimens were produced together in the one build job; however, significant differences in mechanical properties in single sets of specimens, such as maximum reaction force *F_max_*, maximum deformation *x_Dyn_* or duration *t_def_* can be observed (Figure 11b). These differences could be caused by a local damage of the lattice structure under loading, the structure which can occur by the material imperfection of SLM fabricated lattice structures such as surface roughness or internal porosity. It can change symmetrical bending of dominate deformation process, which is typical for BCC structures, to an asymmetrical mechanical response [37]. Therefore, in the case of the lattice structure, it is necessary to work with average values of the mechanical properties. For comparison purposes, the average curves of the force-deformation and initial speed-deformation were created (Figure 11c,d). All the low-velocity impact results are shown in Table 6; there is shown that mechanical properties of sets of specimens, such as maximum reaction *F_max_* and stiffness of the specimens under dynamic loading *k_Dyn_*, increase linearly with struts diameter.

Absorbed energy *E_Abs_* was evaluated regarding the real measured initiating speed *v_In_* and initiating impact energy *E_In_* for each specimen. From Table 5, it is obvious that most of specimens absorbed more than 99% of impact energy, and only in the case of the specimens with nominal diameter *d* = 1.2 mm, there was a small decline. Therefore, the parameter absorption power *P_Abs_* (J·s^−1^), which reflects the deformation and absorbed energy, was defined.

(1) $$P_{Abs}=E_{Abs}/t_{def}$$

The lattice structure with low value of *P_Abs_* can absorb energy through long duration and large deformation. It is important e.g., in automotive industry where the car deformation area must be designed for overload not damaging the human body.

### 3.4. Finite Element Analysis (FEA)

#### 3.4.1. FEA Material Models

Based on the quasi-static results, the material model (BL-I) of the BCC lattice structure from AlSi10Mg alloy was created (Table 7). The parameters *E*, *YTS_0.2%_* and *E_T_* of the TS45-series were used to create the Bilinear isotropic hardening material model due to a similar strut build inclination, as in the case of the BCC lattice structure (35.26°) [33]. A damage criterion was obtained from the C-series as the maximum equivalent plastic strain *ε_σmax_*. The material model (BL-II) of the upper and bottom plate was created using mechanical parameters of the bulk material. The other needed parameters were used from the Ansys material library as the default values.

#### 3.4.2. FEM Model

**Figure 12:**
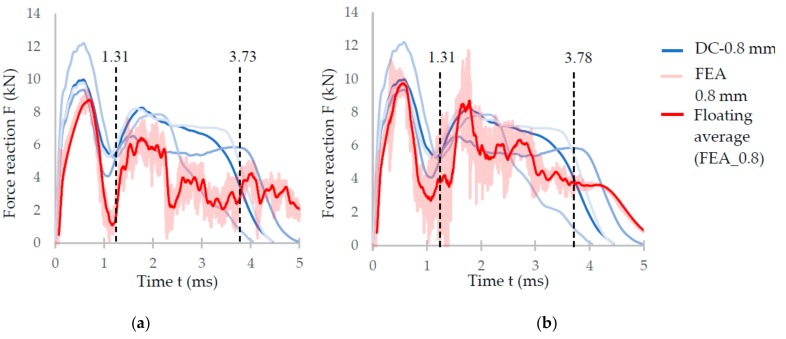
(**a**) Comparison of the results of the IT-0.8 series and the numerical simulation with (**a**) circular cross-section; and (**b**) elliptical cross-section.

**Figure 13:**
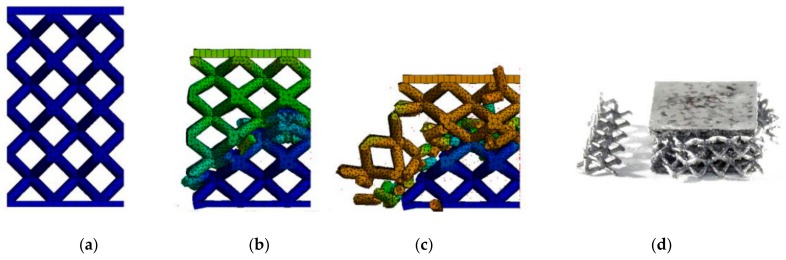
Gradual deformation of the specimen with circular strut cross-section in time—(**a**) 0 ms; (**b**) 1.31 ms; (**c**) 3.73 ms; (**d**) real damage of the specimen IT-2 after low-velocity impact test.

**Figure 14:**
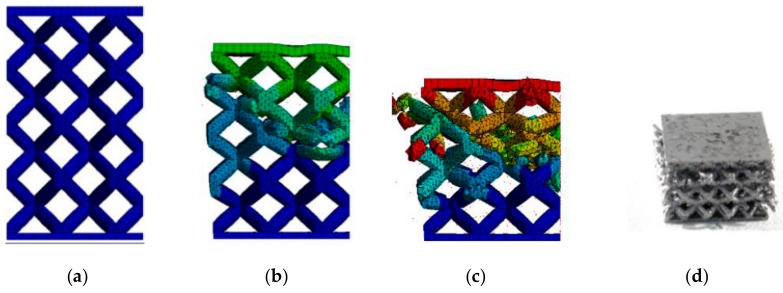
Gradual deformation of the specimen with elliptical strut cross-section in tim: (**a**) 0 ms; (**b**) 1.31 ms, (**c**) 3.78 ms; and (**d**) real damage of the specimen IT-2 after low-velocity impact test.

The results from FEA using the numerical model (NM) of the low-velocity dynamic loading (described above) are shown in Figure 12. From the figure, it is obvious that the force-time curve of the NM with ellipse cross-section (Figure 12b) corresponds better to the experimental results than that with circular cross-section (Figure 12a). The largest deviations can be seen in the middle (between 1.5–4 ms) and towards the end (between 4–5 ms) of the force-time curve. In the case of FEA considering the circular cross-section shape, the deformation time exceeded 5 ms, and the specimen was continually deformed. It does not correspond with the results of the low-velocity testing where the deformation ended at 5 ms. In the case of FEA considering the ellipse cross-section shape, duration and deformation ended at the end of 5 ms. The real and predicted damage of the specimens after low-velocity impact testing is shown in Figure 13 and Figure 14.

The deviations between FEA and the experiment were compared using the maximum force value in the first force peak in the case of FEA, and the average maximum force from the five experimentally tested specimens. The results show that the relative error of FEA with circular cross-section is 12%, while with elliptical cross-section, it is 2% in the case of IT-0.8 series.

## 4. Discussion

### 4.1. Substitution of the Strut’s Real Cross-Section with the Ideal Cross-Section

**Figure 15:**
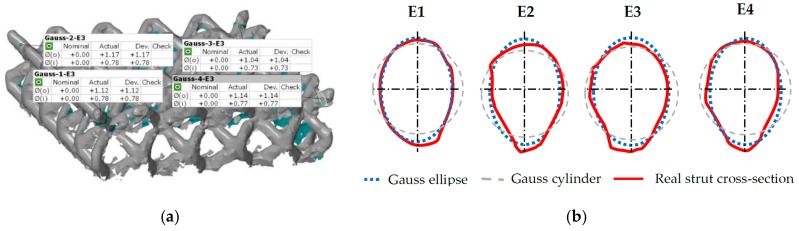
Comparison of the real and ideal cylinder cross-section: (**a**) shape analysis in the GOM Inspect software and (**b**) real cross-section in four corner struts.

The deformation behavior of numerical model (NM) with the ideal circular cross-section geometry of *d* = 0.8 mm (nominal diameter) showed large differences to the experiment during initial tests. Therefore, the results from weighing and optical measuring of the C-series (Figure 15a) were used for finding ideal diameter for using in NM for prediction of the real behavior of the lattice structure.

From the 3D scanned data of the lattice structure(C-series), a cross-section area of the real single strut was calculated (Figure 15b; *A_r_* = 0.712 mm^2^) and compared with the cross-section area of the fitted ideal cylinders to the strut in the GOM Inspect software (*A_Din_* = 0.417 mm^2^; *A_Dgauss_* = 0.701 mm^2^; *A_Dout_* = 1.186 mm^2^). The results show that the best match is in the case of *d_Gauss_*. Therefore, this diameter seems to be appropriate to represent the designed diameter *d* = 0.8 mm in the NM.

A similar result was obtained from weight comparison where the weight of the lattice structure CAD model with *d_Gauss_* and the measured weight were compared (Table 2). To the weight of CAD model (*m_CAD_0.95_*), the larger thickness of the plates from the lighting microscope was also added. The result show that weight *m* and *m_CAD_0.95_* are almost identical. Based on these basic analyses, the strut diameter *d_Gauss_* was selected for lattice structure simplification using ideal circular cross-section in the numerical model. This result differs from the results of Suart et al. [21], where the diameter equal to *d_In_* was used.

During the evaluation of optical measurement, the real shape of the lattice structure struts similar to “water drop” was found (Figure 15b). On the down skin strut surface, surroundings metal powder was melted due to struts orientation and heat transfer [19]. The partially melted powder modifies the strut shape into an elliptical cross-section resulting in an increase of mechanical properties under compression loading (Figure 12). Therefore, if only equivalent circular cross-section is used, the mechanical properties are increase in all directions instead of only Z direction. This will be reflected especially in the FEM model response during the progressive collapse of the lattice structure where deviations from the actual behavior occur, as is shown in Figure 12a. The results of experiment and FEA comparison show that the elliptic cross-section is more suitable for a description of the whole deformation process via FEA (Figure 12b). The circular cross-section can only be used for the estimation of approximate *F_max_* reaction force when the lattice structure starts to be damaged.

### 4.2. Application of Numerical Model to BCC Lattice Structures with Struts Diameter between 0.6–1.2 mm

**Figure 16:**
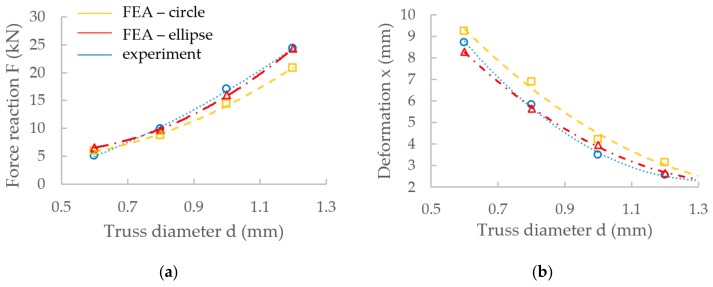
Comparison of FEA results and experiment for different strut diameters; (**a**) reaction force; and (**b**) deformation.

The material model was created directly for the lattice structure with 0.8 mm nominal diameter; therefore, the other specimens, such as those for optical measurement or quasi-static testing, were fabricated only for this nominal diameter. However, as is shown in Figure 16, the material model of the lattice structure can also be used for diameters between 0.6–1.2 mm, which are commonly used dimensions of lattice structure struts.

**Figure 17:**
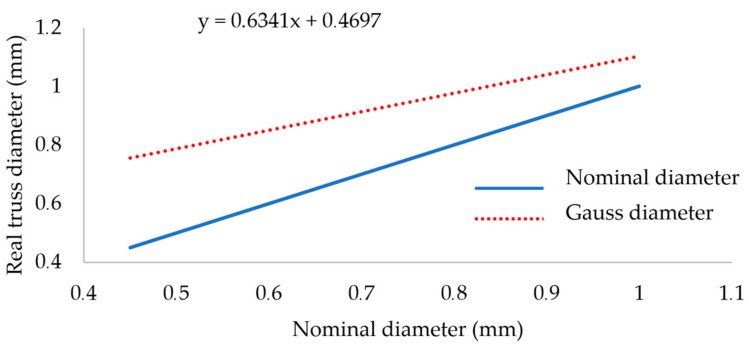
Increase of the real strut diameter fabricated by SLM described in the study [20].

To create the FEM geometry in Ansys software, real strut diameters of nominal diameters 0.6, 1.0 and 1.2 mm were obtained from the previous study [20] where the relation between the designed and real strut diameter after SLM processing was described. In order to use the elliptical shape for these diameters (0.6–1.2 mm), the ellipse ratio *e* from the O-series (*d* = 0.8 mm) was evaluated and applied to other strut sizes using Equation (6). The *d_gauss_* cylinder values from the line equation (Figure 17) [20] were used to calculate the circle cross-section area. Then the elliptical ratio *e* = 0.71 and the equivalent sizes of circular and elliptical cross-sections were used for calculation of minor and major axes of the ellipse. The elliptical ratio was identified as a ratio between the average minor and major ellipse axes in the O-series test. The re-calculation process is described in Equations (2)–(6). The results also confirm a better compliance with the ellipse cross-section than with the circular one (Figure 16).

(2) $$A_{Dgauss}=A_{ellipse}$$

(3) $$\pi \frac{d_{gauss}^{2}}{4}=\pi \cdot a\cdot b$$

(4) $$e=\frac{a}{b}=\frac{0.795/2}{1.114/2}=0.714$$

(5) $$b=\sqrt{d_{gauss}^{2}/4\cdot e}$$

(6) a=e·b

### 4.3. Mechanical Testing

In their study [34], the Tsopanos et al. tested single struts of 316L with diameters of about 0.2 mm. The mechanical properties of struts were half as compared to the standard material because the mechanical properties of a single strut mainly decrease porosity and surface roughness. From this, it follows that to find the correct mechanical properties for the numerical model of lattice structure, it is not suitable to use the bulk material tensile specimens.

Nevertheless, during compression loading, a lot of single struts transfer the load in the lattice structure. Therefore, multi-strut tensile specimen, where more struts are also loaded simultaneously were designed. The results of tensile testing show that specimens fabricated by SLM with of 45° orientation have different mechanical properties in comparison with those of 90° orientation − *YTS_0.2%_* + 10%; *UTS* + 20%; *E* + 40%; and *E_t_* − 30%. It could be due to a higher porosity level inside the strut in the case of 90° orientation. To obtain the correct mechanical properties during evaluation of strut mechanical properties, it is necessary to use the real dimensions measured e.g., by optical measurement. The strut mechanical properties were compared with bulk material which is not too affected by internal defects. The results show much lower strut mechanical properties and more brittle material. (*YTS_0.2%_* − 40%; *UTS* − 30%; Young’s modulus *E* − 30% and Tangent modulus *E_t_* + 30 ÷ 50%). It may be mainly caused by significant surface roughness and almost two times higher surface of multi-strut specimens compare to bulk specimens (970/565 mm^2^, calculated using Gaussian diam. for specimens T45-series *d_Gauss_* = 0.89 mm.). The size of specimen’s surface is also connected with close to surface porosity which can be expressed using parameter *CtS* and Equation (7) (for one truss of multi-strut spec., it is of 130; for bulk spec., it is of 29). This parameter expresses the ratio between the surface of the specimen or struts in multi-strut specimen *S* (mm^2^) and cross-section of the specimen or strut *A* (mm^2^). Its value shows susceptibility to failure due to close to surface porosity.

(7) $$CtS=\frac{S}{A}=\frac{n\cdot \pi \cdot d\cdot h_{ef}}{n\cdot \pi \frac{d^{2}}{4}}=\frac{4h_{ef}}{d}=28.8$$

where *n* is number of the struts of the specimens (for bulk shape *n* = 1), *d* is the strut or bulk specimens’ diameter and *h* is the effective area of the specimen (see Figure 3).

### 4.4. Criterion of Damage

A damage criterion is the Ansys parameter which defines when the element is excluded from calculation (element erosion) and no longer contributes to load transfer. In the case of presented numerical model, the Equivalent Plastic Strain *EPS* = 0.1025 was used (Table 6). It means that if the element is deformed more than 10.25%, it is removed.

The true strain value at the area of the damage of tensile specimen is required as input for this criterion in Ansys. From the strut tensile testing, only the global specimen’s strain without considering the local damage in the critical area was obtained. There are two reasons: Firstly, it was an atypical shape of the specimens where it was problematic to measure the narrowing of the single struts in the damaged area. Secondly, the used material is very brittle; therefore, the narrowing of the struts was very small and could not be measured with available equipment. For this reason, an alternative method was used; *EPS* was represented by the strain at the first peak *F_max_* in the compression test.

## 5. Conclusions

In this study, all processes of material model creation and final FEA analysis were presented. The results show that the SLM technology allows to produce energy absorbers from AlSi10Mg alloy, which can effectively absorb energy through self-deformation. Due to a good accordance between the numerical model and the experiment, it was possible to use the numerical model of lattice structure for precise design of the absorber in high-performance applications. This model will be used for future testing of geometry changes and their impact on mechanical properties. The presented process of finding the material model can be employed for various materials used for SLM production.

- The numerical model of BCC micro-lattice structure under dynamic loading with the elliptic strut shape was developed. The results show that the elliptic shape of the lattice structure significantly decreases a deviation between FEA and the measured results compared to the circular cross-section (10%, measured in the first force peak).
- To find the correct mechanical properties for FEA material model, it is necessary to use the struts specimens with appropriate orientation during production due to the influence of internal porosity and surface roughness.
- The orientation during SLM production significantly influences the mechanical properties.
- The shape of the BCC lattice structure was analyzed using optical methods. A distinct “water drop” shape was found in the case of AlSi10Mg alloy.
- A weight comparison of the CAD design and the produced lattice structure shows that for simplification of the “water drop” shape of the strut, the Gaussian strut diameter should be used.
- The results of quasi-static mechanical testing show that the differences between mechanical properties of the 90° and 45° orientation are mainly in the plastic area of deformation and may by caused by the significant surface roughness.

## Figures and Tables

**Table 1:** The list of used abbreviation.

Shortcut	Description	Shortcut	Description
*SLM*	Selective laser melting technology	*d_in_*	Maximum inscribed cylinder into the strut
*FEA*	Finite element analysis	*d_out_*	Minimum circumscribed cylinder on the strut surface
*FEM*	Finite element method	*A_r_*	Cross-section area of real strut
*YLR*	Ytterbium fiber laser	*A_Din_*	Cross-section area of maximum inscribed cylinder into a strut
*BCC*	Body centered cubic	*A_Dgauss_*	Cross-section area of Gauss strut cylinder
*NM*	Numerical model	*A_Dout_*	Cross-section area of minimum circumscribed cylinder fitted on a strut surface
*STF*	Shell thickness factor	*A_ellipse_*	Cross-section area of an ellipse fitted to the strut surface
*CAD*	Computer aided design	*a*	Ellipse minor axes
*EPS*	Equivalent Plastic Strain	*b*	Ellipse major axes
*BL-I*	Bilinear isotropic hardening model of lattice core	*e*	Ellipse ratio
*BL-II*	Bilinear isotropic hardening model of bottom and upper plates	*F_max_*	Maximum force
*EBM*	Electron beam melting	*x_Fmax_*	Deformation of the specimen at maximum force
*CT*	Computed tomography	*σ_max_*	Maximum engineering stress
*a_BCC_*	Length of BCC cell edge	*ε_σmax_*	Strain at the maximum engineering stress
*l*	Length of the struts in the multi-strut tensile specimen	*E*	Young’s Modulus
*d*	Nominal lattice structure strut diameter	*E_T_*	Tangent Modulus
*t*	Specimen’s upper plate thickness	*YTS_0.2%_*	Offset yield strength at strain 0.2%
*h*	Height of the C-series specimens	*UTS*	Ultimate tensile strength
*h_CAD_*	Nominal CAD height of the specimen	*E_In_*	Initiating impact energy, energy just before impact
*t_UpP_*	Thickness of the upper plate	*v_In_*	Initiating speed, speed just before impact
*m_C_*	Weight of the C-series specimens	*m*	Weight of the falling head
*m_CAD_0.8_*	CAD weight of the C-series specimen with nominal struts dimeter	*t_def_*	Duration of deformation
*m_CAD_0.95_*	CAD weight of the C-series specimen with Gauss stuts diameter and real upper plate thickness	*x_Dyn_*	Deformation of the specimens under dynamic loading
$\bar{\rho }$	Measured relative density of C-series	*E_Abs_*	Absorbed energy
$\bar{\rho }$ *_CAD_0.8_*	Calculated relative density of the CAD model with nominal diameter *d* = 0.8 mm	*v_Up_*	Speed of the rebound
$\bar{\rho }$ *_CAD_0.95_*	Calculated relative density of the CAD model with measured Gaussian diameter *d* = 0.8 mm	*k_Dyn_*	Average stiffness of the specimens under dynamic loading
*d_gauss_*	Ideal struts Gauss cylinder	*P_Abs_*	Absorption power of the specimens under dynamic loading
*n*	Number of the struts in the multi-strut specimen	*h_ef_*	Effective length of the tensile specimen
*ρ_Ind_*	deliberately increased density of the indenter to represent the weight of the whole falling head	*E_inp_*	Input energy to the current layer of the lattice structure
*SEM*	Scanning electron microscopy	*E_lin_*	Linear energy—(laser power/laser speed)

**Table 2:** The initial analysis of the C-series.

(Avg. Values)	Measured					CAD			
	*h*	*t_UpP_*	*m*	$\bar{\rho }$	*h_CAD_*	*m_CAD_0.8_*	*m_CAD_0.95_*	$\bar{\rho }$ *_CAD_0.8_*	$\bar{\rho }$ *_CAD_0.95_*
	(mm)	(mm)	(g)	(%)	(mm)	(g)	(g)	(%)	(%)
$\bar{x}$	21.04	0.75	6.97	31	20.80	4.72	6.94	21	31

**Table 3:** Struts diameter measured using the Atos Triple Scan optical system (O-series; nominal diameter *d* = 0.8 mm).

(mm)	Corner Strut	*d_gauss_*		*d_in_*		*d_out_*		Ellipse			
								Minor Axis		Major Axis	
**S1**	1	0.94	0.95	0.74	0.73	1.26	1.21	0.79	0.79	1.1	1.12
	2	0.99		0.75		1.19		0.81		1.17	
	3	0.93		0.7		1.24		0.79		1.14	
	4	0.93		0.72		1.16		0.78		1.09	
**S2**	1	0.96	0.96	0.76	0.74	1.18	1.22	0.8	0.79	1.2	1.12
	2	0.92		0.75		1.09		0.79		1.03	
	3	1.02		0.73		1.36		0.8		1.06	
	4	0.94		0.72		1.23		0.77		1.17	
**S3**	1	0.86	0.91	0.69	0.71	1.08	1.18	0.78	0.76	1.08	1.06
	2	0.91		0.69		1.26		0.77		1.05	
	3	0.94		0.76		1.2		0.76		1.13	
	4	0.91		0.7		1.17		0.73		0.97	
**S4**	1	0.97	0.97	0.82	0.74	1.27	1.31	0.86	0.84	1.27	1.16
	2	0.96		0.73		1.31		0.89		1.15	
	3	1.01		0.74		1.43		0.83		1.04	
	4	0.93		0.67		1.23		0.77		1.18	
$\bar{x}$		0.945		0.729		1.229		0.795		1.114	

**Table 4:** The dimensions of the tensile specimen specimens (multi-struts tensile specimens TS-series; bulk tensile specimens TB-series).

(mm)	TS45			TS90			TB45			TB90		
	*d_gauss_*	*d_in_*	*d_out_*	*d_gauss_*	*d_in_*	*d_out_*	*d_gauss_*	*d_in_*	*d_out_*	*d_gauss_*	*d_in_*	*d_out_*
1	0.88	0.66	1.07	0.78	0.61	1.09	5.05	4.91	5.49	5.03	4.94	5.36
2	0.88	0.69	1.14	79	0.68	1.03	5.04	4.89	5.66	5.02	4.9	5.45
3	0.89	0.72	1.15	-	-	-	5.03	4.85	5.6	5.01	4.93	5.57
4	0.9	0.74	1.19	0.79	0.71	0.88	-	-	-	-	-	-
5	0.9	0.7	1.34	0.8	0.69	1.06	-	-	-	-	-	-
6	0.91	0.71	1.29	0.78	0.69	0.87	-	-	-	-	-	-
$\bar{x}$	0.89	0.70	1.20	0.79	0.68	0.99	5.04	4.88	5.58	5.02	4.92	5.46

**Table 5:** The dimensions of the tensile specimen specimens with different orientation to the platform (multi-struts tensile specimens TS-series; bulk tensile specimens TB-series).

*Spec.*	*F_max_* (N)	*x_Fmax_* (mm)	*σ_max_* (MPa)	*ε_σmax_*	*E* (GPa)	*YTS_0.2%_* (MPa)	*UTS* (MPa)	*E_T_* (MPa)
TS45	2270	0.462	-	0.015	71.6	131.6	224.2	6649
TS90	1934	0.297	-	0.010	103.7	116.6	186.8	8701
TB45	7625	1.030	-	0.026	96.1	227.0	382.2	4858
TB90	6453	0.809	-	0.020	147.5	187.4	326	5753.3
C	10,860	2.133	27.2	0.103	483.5	-	-	-

**Table 6:** The results of the low-velocity impact.

#	*F_max_* (N)	*t_def_* (ms)	*x_Dyn_* (mm)	*v_In_* (m·s^−1^)	*E_In_* (J)	*E_Abs_* (J)	*v_Up_* (m·s^−1^)	*k_Dyn_* (N·mm^−1^)	*P_Abs_* (J·s^−1^)
IT 0.6	4252	4.94	9.07	3.02	33.10	32.47	0.42	9005	6.58
	6479	4.64	7.67	2.95	31.51	31.19	0.30		6.73
	4005	5.29	9.61	2.93	31.19	30.87	0.30		5.83
	4660	5.04	8.86	2.95	31.48	31.20	0.28		6.19
	6047	4.71	8.31	2.97	32.08	31.68	0.33		6.73
$\bar{x}$	5089	4.92	8.70	2.96	31.87	31.48	0.32	-	6.41
IT 0.8	-	-	-	-	-	-	-	-	-
	9989	3.41	5.15	2.97	32.03	31.58	0.35	19,417	9.27
	9368	4.05	6.00	2.93	31.91	31.71	0.24		7.82
	12,218	2.94	4.32	>2.96	31.87	31.31	0.39		10.66
	9795	3.52	5.43	2.96	31.72	31.08	0.42		8.83
$\bar{x}$	10,343	3.48	5.22	2.96	31.88	31.42	0.35	-	9.15
IT 1.0	15,223	2.79	3.83	3.07	34.22	33.89	0.30	29,371	12.14
	17,625	2.03	3.30	3.13	35.45	35.28	0.22		17.37
	16,437	2.16	3.66	3.15	36.09	35.56	0.38		16.49
	18,796	1.80	3.08	3.16	36.09	35.29	0.47		19.58
	16,859	2.18	3.50	3.15	35.98	35.83	0.20		16.46
$\bar{x}$	16,988	2.19	3.47	3.13	35.57	35.17	0.31	-	16.41
IT 1.2	24,205	1.49	2.43	3.19	36.93	34.87	0.75	39,006	23.41	
	28,067	1.31	2.17	3.22	37.61	35.22	0.81		26.84
	20,597	1.89	3.14	3.21	37.30	36.44	0.48		19.33
	27,627	1.31	2.13	3.21	37.28	34.92	0.81		26.61
	20,990	1.80	2.87	3.17	36.54	35.41	0.56		19.65
$\bar{x}$	24,297	1.56	2.55	3.20	37.13	35.38	0.68	-	23.17

**Table 7:** Materials model used for lattice structure specimens FEA.

Parameters	BL-I (BCC)	BL-II (Plate)	Unit
Density	2680	2680	kg·m^−3^
Isotropic Elasticity	-	-	-
Young’s Modulus	70,723	96,100	MPa
Poisson’s Ratio	0.334	0.334	-
Bulk Modulus	7.1 × 10^10^	9.6 × 10^10^	Pa
Shear Modulus	2.7 × 10^10^	3.6 × 10^10^	Pa
Bilinear Isotropic Hardening	-	-	-
Yield Strength	135	227	MPa
Tangent Modulus	6586	4858	MPa
Plastic Strain Failure	-	-	-
Max. Equivalent Plastic Strain EPS	0.1025	0.1025	-

## References

[1] Karagiozova D. (2004). Dynamic buckling of elastic-plastic square tubes under axial impact—I: Stress wave propagation phenomenon. Int. J. Impact Eng..

[2] Li X., Zhang P., Wang Z., Wu G., Zhao L. (2014). Dynamic behavior of aluminum honeycomb sandwich panels under air blast: Experiment and numerical analysis. Compos. Struct..

[3] Olabi A.G., Morris E., Hashmi M.S.J. (2007). Metallic tube type energy absorbers: A synopsis. Thin Walled Struct..

[4] Dharmasena K.P., Wadley N.G., Xue Z., Hutchinson J.W. (2008). Mechanical response of metallic honeycomb sandwich panel structures to high-intensity dynamic loading. Int. J. Impact Eng..

[5] Kopanidis A., Theodorakakos A., Gavaises E., Bouris D. (2010). 3D numerical simulation of flow and conjugate heat transfer through a pore scale model of high porosity open cell metal foam. Int. J. Heat Mass Transf..

[6] Shimizu T., Matsuzaki K., Nagai H., Kanetake N. (2012). Production of high porosity metal foams using EPS beads as space holders. Mater. Sci. Eng. A.

[7] Zhu L., Guo K., Li Y., Yu T.X., Zhou Q. (2018). Experimental study on the dynamic behaviour of aluminium foam sandwich plates under single and repeated impacts at low temperature. Int. J. Impact Eng..

[8] Sun B., Zhang R., Zhang Q., Gideon R., Gu B. (2013). Drop-weight impact damage of three-dimensional angle-interlock woven composites. J. Compos. Mater..

[9] Vrana R., Koutny D., Paloušek D. (2016). Impact Resistance of Different Types of Lattice Structures Manufactured by SLM. MM Sci. J..

[10] Mines R.A.W., Tsopanos S., Shen Y., Hasan R., McKown S.T. (2013). Drop weight impact behaviour of sandwich panels with metallic micro lattice cores. Int. J. Impact Eng..

[11] Harris J.A., Winter R.E., McShane G.J. (2017). Impact response of additively manufactured metallic hybrid lattice materials. Int. J. Impact Eng..

[12] Yadroitsev I. (2009). Selective Laser Melting: Direct Manufacturing of 3D-Objects by Selective Laser Melting of Metal Powders.

[13] Thijs L., Kempen K., Kruth J.-P., van Humbeeck J. (2013). Fine-structured aluminium products with controllable texture by selective laser melting of pre-alloyed AlSi10Mg powder. Acta Mater..

[14] Qiu C., Yue S., Adkins N.J.E., Ward M., Hassanin H., Lee P.D., Withers P.J., Attallah M.M. (2015). Influence of processing conditions on strut structure and compressive properties of cellular lattice structures fabricated by selective laser melting. Mater. Sci. Eng. A.

[15] Koutny D., Palousek D., Pantelejev L., Hoeller C., Pichler R., Tesicky L., Kaiser J. (2018). Influence of scanning strategies on processing of aluminum alloy EN AW 2618 using selective laser melting. Materials.

[16] Han X., Zhu H., Nie X., Wang G., Zeng X. (2018). Investigation on Selective Laser Melting AlSi10Mg Cellular Lattice Strut: Molten Pool Morphology, Surface Roughness and Dimensional Accuracy. Materials.

[17] Ilcik J., Koutny D., Palousek D. Geometrical accuracy of the metal parts produced by selective laser melting: Initial tests. Proceedings of the 54th International Conference of Machine-Design-Departments (ICMD).

[18] Skalicky P., Koutny D., Pantelejev L., Palousek D. Processing of aluminum alloy EN AW 7075 using selective laser melting: Initial study. Proceedings of the 58th International Conference of Machine-Design-Departments (ICMD2017).

[19] Vrana R., Koutny D., Palousek D., Pantelejev L., Jaros J., Zikmund T., Kaiser J. (2018). Selective laser melting strategy for fabrication of thin struts usable in lattice structures. Materials.

[20] Koutny D., Vrana R., Paloušek D. Dimensional accuracy of single beams of AlSi10Mg alloy and 316L stainless steel manufactured by SLM. Proceedings of the 5th International Conference on Additive Technologies (iCAT2014).

[21] Suard M., Lhuissier P., Dendievel R., Vignat F., Blandin J.J., Villeneuve F. Impact of EBM fabrication strategies on geometry and mechanical properties of titanium cellular structures. Proceedings of the Fraunhofer Direct Digital Manufacturing Conference (DDMC 2014).

[22] Vrana R., Koutny D., Paloušek D., Zikmund T. Influence of selective laser melting process parameters on impact resistance of lattice structure made from AlSi10Mg. Proceedings of the World PM 2016 Congress and Exhibition.

[23] Grytten F., Børvik T., Hopperstad O.S., Langseth M. (2009). Low velocity perforation of AA5083-H116 aluminium plates. Int. J. Impact Eng..

[24] Grytten F., Holmedal B., Hopperstad O.S., Børvik T. (2008). Evaluation of identification methods for YLD2004-18p. Int. J. Plast..

[25] Mohmmed R., Ahmed A., Elgalib M.A., Ali H. (2014). Low Velocity Impact Properties of Foam Sandwich Composites: A Brief Review. Int. J. Eng. Sci. Innov. Technol..

[26] Mohmmed R., Zhang F., Sun B., Gu B. (2013). Finite element analyses of low-velocity impact damage of foam sandwiched composites with different ply angles face sheets. Mater. Des..

[27] Labeas G., Ptochos E. (2013). Investigation of sandwich structures with innovative cellular metallic cores under low velocity impact loading. Plast. Rubber Compos..

[28] Ozdemir Z., Tyas A., Goodall R., Askes H. (2017). Energy absorption in lattice structures in dynamics: Nonlinear FE simulations. Int. J. Impact Eng..

[29] Banerjee A., Dhar S., Acharyya S., Datta D., Nayak N. (2015). Determination of Johnson cook material and failure model constants and numerical modelling of Charpy impact test of armour steel. Mater. Sci. Eng. A.

[30] Milani A.S., Dabboussi W., Nemes J.A., Abeyaratne R.C. (2009). An improved multi-objective identification of Johnson-Cook material parameters. Int. J. Impact Eng..

[31] Brandl E., Heckenberger U., Holzinger V., Buchbinder D. (2012). Additive manufactured AlSi10Mg samples using Selective Laser Melting (SLM): Microstructure, high cycle fatigue, and fracture behavior. J. Mater. Des..

[32] Vaverka O., Koutný D., Vrána R., Pantělejev L., Paloušek D. Effect of heat treatment on mechanical properties and residual stresses in additively manufactured parts. Proceedings of the Engineering Mechanics 2018 24th International Conference.

[33] Kempen K., Thijs L., van Humbeeck J., Kruth J.-P. (2012). Mechanical properties of AlSi10Mg produced by selective laser melting. Phys. Procedia.

[34] Tsopanos S., Mines R.A.W., Mckown S., Shen Y., Cantwell W.J., Brooks W., Sutcliffe C.J. (2010). The influence of processing parameters on the mechanical properties of selectively laser melted stainless steel microlattice structures. J. Manuf. Sci. Eng..

[35] Xiao L., Song W. (2018). Additively-manufactured functionally graded Ti-6Al-4V lattice structures with high strength under static and dynamic loading: Experiments. Int. J. Impact Eng..

[36] Palousek D., Omasta M., Koutny D., Bednar J., Koutecky T., Dokoupil F. (2015). Effect of matte coating on 3D optical measurement accuracy. Opt. Mater..

[37] Ravari M.R.K., Kadkhodaei M., Ghaei A. (2016). Effects of asymmetric material response on the mechanical behavior of porous shape memory alloys. J. Intell. Mater. Syst. Struct..

